# P-234. Analyzing Robustness and Frailty: Musculoskeletal Injuries, Fractures, and Hospitalizations in U.S. Military Members with and without HIV

**DOI:** 10.1093/ofid/ofaf695.456

**Published:** 2026-01-11

**Authors:** Brenna M Roth, Mackensie Horn, Jacqueline M Causbie, Senay Topal, Anuradha Ganesan, Daniel Clifton, Robert O’Connell, Brian Agan

**Affiliations:** Henry M. Jackson Foundation for the Advancement of Military Medicine, Baltimore, Maryland; Infectious Disease Clinical Research Program, Bethesda, Maryland; Walter Reed National Military Medical Center; 4Uniformed Services University of the Health Sciences, North Bethesda, MD; Uniformed Services University of Health Sciences, Bethesda, Maryland; Consortium for Health and Military Performance, Uniformed Services University; Henry M. Jackson Foundation for the Advancement of Military Medicine, Inc., Bethesda, Maryland; Infectious Disease Clinical Research Program, USUHS, Bethesda, Maryland; Infectious Disease Clinical Research Program, Department of Preventive Medicine and Biostatistics, Uniformed Services University of the Health Sciences, Bethesda, MD, USA, Bethesda, Maryland

## Abstract

**Background:**

People with HIV (PWH) accumulate more comorbidities and are frailer at a younger age compared to people without HIV (PWoH). The Kim Index uses claims-based medical codes from electronic medical records to identify robustness and frailty, mostly in older people, and predict outcomes relevant to them. Musculoskeletal injuries (MSKI) and fractures are common and relevant outcomes in the active duty service member (ADSM) population. We evaluated the association of Kim Index scores with these outcomes and hospitalizations among ADSM PWH and PWoH.

**Table 1:**
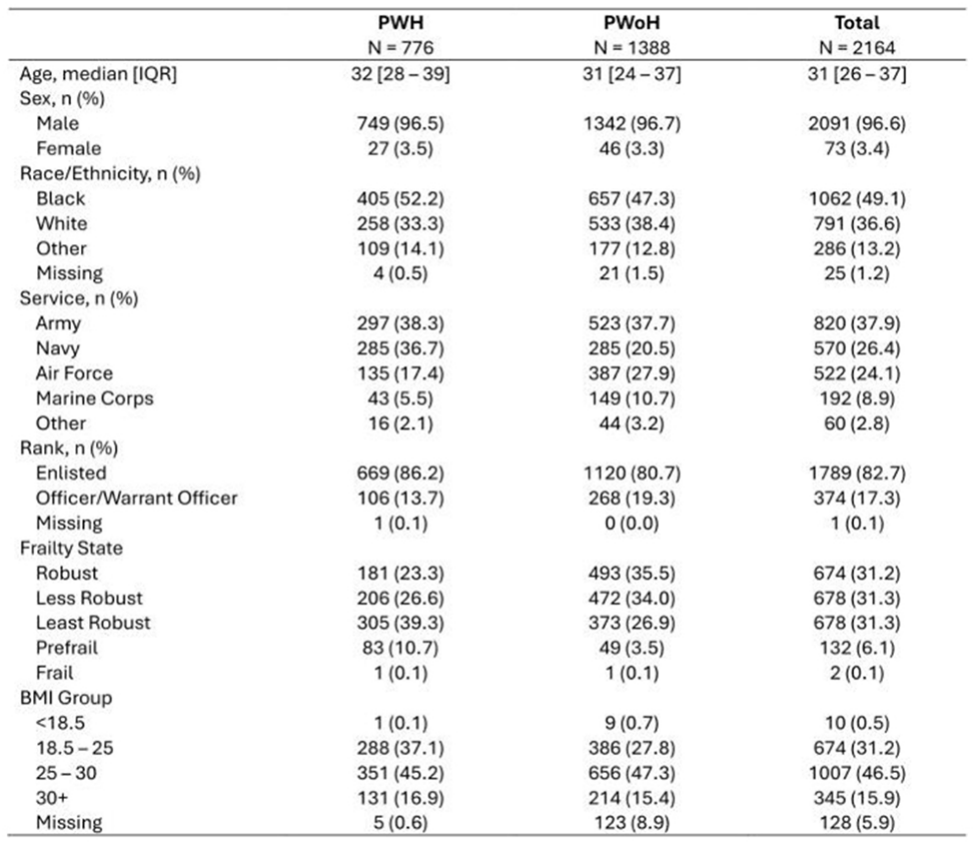
Baseline characteristics of active duty service military in 2014

**Figure 1:**
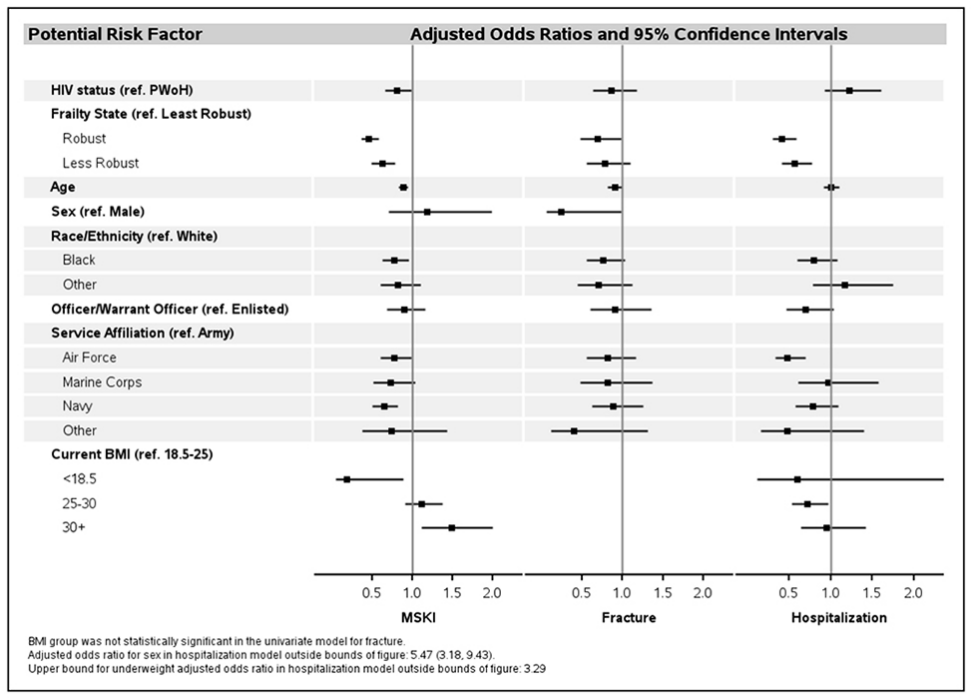
Multivariate analysis of factors associated with musculoskeletal injuries, fractures, and hospitalization

**Methods:**

We analyzed data from the HIV Virtual Cohort Study, a retrospective, longitudinal cohort study of DoD beneficiary PWH and PWoH. We used data from 2014 to 2018 and logistic regression models to evaluate the association of robustness and other factors with the outcomes of MSKI, fracture, and hospitalization. Baseline characteristics were described for 2014 and evaluated for the odds of developing the outcomes within 5 years.

**Results:**

The baseline median age was 31 (IQR 26-37), with the largest proportion men (96.6%), black (49.1%), in the Army (37.9%), and enlisted (82.7%). The Kim Index classified 89.2% of PWH and 96.4% of PWoH as robust. The largest proportion of PWH and PWoH were least robust (39.2%) and robust (35.5%) respectively. In multivariate analysis, a lower odds of MSKI was associated with HIV, robust or less robust, increased age, Air Force or Navy service, and a BMI < 18.5. A higher odds of MSKI was associated with a BMI >30. A lower odds of fracture was associated with being robust and female. A lower odds of hospitalization was associated with robust or less robust, Air Force service, and a BMI of 25-30. A higher odds of hospitalization was associated with female.

**Conclusion:**

ADSM PWH and PWoH are largely robust as defined by the Kim Index. Our analysis indicates differences in the odds of MSKI, fracture, and hospitalization in least robust compared to less robust and robust; however, HIV appears to be protective in the case of MSKI. This may be due to the historical exclusion of HIV infected people from combat job positions. Discriminating within the category of robust may identify subgroups in this population at greater risk for relevant outcomes. Further evaluation of risk determinants is ongoing to understand the unexpected HIV findings.

**Disclosures:**

All Authors: No reported disclosures

